# Efficacy of methenamine with methylthioninium in the treatment of dysuria: a randomized clinical study

**DOI:** 10.1007/s00192-023-05669-0

**Published:** 2023-10-18

**Authors:** Felício Savioli Neto, Helena Hachul, Márcio Antônio Pereira, Carlos Isaia Filho

**Affiliations:** 1 Dr. Felício Savioli Neto Cardiologia e Cardiogeriatria, Cotia, SP Brazil; 2 Department of Psicobiology, Universidade Federal de São Paulo, São Paulo, SP Brazil; 3 ISPEM – Instituto São José dos Campos em Pesquisas Médicas, São José dos Campos, SP Brazil; 4 Centro de Medicina Reprodutiva Dr. Carlos Isaia Filho, Rua Hilário Ribeiro 202/304, Porto Alegre, RS Brazil

**Keywords:** Dysuria, Pain, Urinary tract infections, Methenamine, Non-antibiotic strategies

## Abstract

**Introduction and hypothesis:**

Dysuria is a common symptom present in several urological and gynecological conditions. Management relies on the underlying disease but may require additional symptomatic treatment. This study evaluated the combination of methenamine 250 mg and methylthioninium chloride 20 mg in the treatment of dysuria versus phenazopyridine.

**Methods:**

This was a multicenter, single-blind, randomized, superiority clinical trial, including individuals over 18 with dysuria and a score ≥ 5 points on the pre-treatment categorical scale for pain. The primary outcome was the proportion of participants presenting excellent clinical response within 24 h after treatment. Improvement up to 72 h, time to reach improvement, sustained healing, investigators’ opinion, and safety were also evaluated.

**Results:**

Three hundred and fifteen participants were evaluated. Demographic characteristics and symptoms of dysuria were comparable between groups at baseline. The difference in the excellent response rate between treatments within 24 h was 12.7% (95% CI 6.16, 19.21) for pain, 9.4% (95% CI 3.32, 15.39) for burning, and 12.7% (95% CI 6.37, 18.99) for burning on urination, all in favor of the test drug, which was also superior from 36 to 48 h. Treatments were similar concerning time to reach the absence of symptoms and in the percentage of participants with sustained healing after 72 h.

**Conclusions:**

The association of methenamine with methylthioninium is superior to phenazopyridine in the treatment of dysuria.

## Introduction

Dysuria is a symptomatic manifestation of several urological and gynecological diseases [1], characterized by a sensation of pain, burning, or stinging on urination [2]. Around 3% of adults over 40 have dysuria, causing distress [3], but a higher prevalence may be observed depending on the underlying condition. The most frequent cause of acute dysuria is urinary tract infections (UTI) [2], affecting about 12% of adult women each year, especially those under 30 [4]. UTI is responsible for almost 40% of cases in emergency departments (ED) in Brazil [5] and more than 1 million visits to ED per year in the United States [6]. Still, any inflammation or irritation of the urethra or bladder can cause dysuria, such as urethritis, sexually transmitted diseases, stones, tumors, food, medications, and menopause [2].

Management of dysuria relies on the cause; discomfort relief may require additional symptomatic treatment. Phenazopyridine, formerly known as an antiseptic, is an analgesic widely used in urinary tract symptoms [7]. Still, limited evidence on efficacy and especially concerns about its safety [7] create a demand for other available choices. Methenamine hippurate and methylthioninium chloride have antiseptic and anti-inflammatory properties; they have been used in the prophylaxis of UTIs with a low risk of drug resistance [8–10]. However, to our knowledge, comparison with phenazopyridine and assessment of dysuria not limited to UTI had not been carried out before.

In this study we hypothesized that the combination of methenamine 250 mg and methylthioninium chloride 20 mg is superior to phenazopyridine in the treatment of dysuria.

## Materials and methods

### Study design and ethics

This was a phase III, prospective, multicenter, single-blind, randomized, superiority clinical trial, conducted in four research centers in Brazil, between June 2016 and December 2017. The study was carried out in compliance with Good Clinical Practice, the International Council for Harmonization, and the Declaration of Helsinki and its amendments. The study protocol was approved by an independent local ethics committee (number 1.040.734), registered on clinicaltrials.gov (NCT01657448), and all participants gave their written consent before entering into the study. Guidelines from the Consolidated Standards of Reporting Trials (CONSORT) were followed.

### Study population

Eligible criteria were participants of both sexes, aged 18 years and older, presenting dysuria with scores ≥ 5 points on the pre-treatment categorical scale for the pain symptom (seven-point scale: 1–2: absent; 3–4: mild; 5–6: moderate; 7: severe). Women of childbearing age and who were sexually active had to be using safe contraceptive methods and had to present a negative pregnancy test before inclusion.

Participants were not included if they were pregnant or breastfeeding, had a history of sensitivity to any of the components of the test drug or comparator, presented fever (axillary T°: ≥ 38.5ºC) with dorsal or lumbar pain, kidney stones, urethral stricture, primary kidney disease, neurogenic bladder, or complicated UTI. Other exclusion criteria were deficiency of the enzyme glucose-6-phosphate-dehydrogenase, severe dehydration, metabolic acidosis or gout, pyelonephritis, use of serotonergic or antimicrobial drug within 7 days before the beginning of the study. Participants were also not included if they had any condition that prevented them from participating in the study because of health-related problems (epilepsy, depression, severe liver or renal dysfunction) or according to the investigators’ evaluation, as well as those who had participated in clinical study protocols in the last 12 months.

### Randomization, allocation, and blinding

Participants were randomized using a sequence generated by the Excel program with a blocking scheme in a 1:1 ratio, and treatment was available as per the randomization list. A biostatistician-validated randomization list of masked treatments was provided, and the allocation of the research participants was carried out sequentially in the order of inclusion in the study until the final number of evaluable participants was reached. To ensure blinding, the sponsor was responsible for randomization and distribution, and the medication was dispensed to the participant by a professional chosen by the principal investigator. Additionally, participants were instructed not to report to the investigators changes in urinary color or any product characteristic.

### Intervention

Group 1 received the investigational drug, consisting of the combination of methenamine 250 mg and methylthioninium chloride 20 mg (EMS, Hortolândia, Brazil), two tablets by oral route every 8 h for 3 days. Group 2 received the comparator, consisting of phenazopyridine hydrochloride 100 mg (Pyridium®, Zodiac), two pills via the oral route every 8 h for 3 days.

Laboratory tests were carried out at the beginning and the end of the study and comprised blood count, biochemistry, urine analysis, and urine culture with antibiogram. Participants had two visits to the research center: V1 (beginning of treatment) and V2 (4 days ± 1). Adjuvant therapy was acceptable in the case of urinary bacterial infection confirmed by urine culture, with ciprofloxacin 250 mg (Proflox®; EMS) twice a day for 3 days, given at V2, and two extra visits were required: V3 (7 days ± 1) and V4 (12 ± 1). Those who used an analgesic during the study were excluded from the trial, and their data were analyzed only for safety.

### Outcomes

The primary outcome was the improvement of dysuria, evaluated as the percentage of participants presenting an excellent clinical response, defined as an improvement in symptoms from mild, moderate, or intense to absent, from 0 to 24 h after treatment.

Dysuria was divided into three components: pain, burning, and burning on urination, and participants used the categorical seven-point scale and a 100-mm visual analog scale (“0” = absence of the symptom and “100” = maximum intensity) to report symptoms.

The secondary outcomes were:

1. Percentage of participants with an excellent clinical response at 12 h, 36 h, 48 h, and 72 h
2. Time to reach absence of symptoms
3. Sustained healing—defined as the absence of pain after 72 h
4. Improvement of dysuria by the investigator using the Clinical Global Impression (CGI) scale, which consisted of two subscales, the severity (CGI-S) and the improvement (CGI-I) [11]. The principal investigator evaluated the final effectiveness after clinical examination and analysis of the pre- and post-treatment scores duly completed and classified based on the subscale CGI-I, which measures the individual's global clinical improvement from baseline and ranges from 1 (very much improved) to 7 (very much worse)
5. Urine culture at the end of the treatment
6. Safety, by the incidence of adverse events (AEs)

### Sample size calculation

The sample size was calculated using a parallel design with two samples and a superiority test for proportions. We adopted a superiority margin of 1.85% based on studies evaluating the improvement of dysuria of phenazopyridine hydrochloride and placebo [12–15]. To reach a power of 80%, with a significance level of 5%, and the superiority margin of 1.85%, the number of participants needed to detect the difference between groups was 274. However, considering a 15% drop-out rate, the total sample was 316 participants, 158 in each treatment group.

### Statistical analysis

The analysis was carried out using the intention-to-treat (ITT) population, evaluating all participants who were included in the study after randomization, who received at least one dose of the medication, and who presented at V2.

Demographic data were evaluated using descriptive analysis, presenting mean, median, standard deviation, minimum, and maximum if necessary.

We calculated the percentage of participants presenting an excellent clinical response in 24 h for each group with the 95% confidence interval (CI), and the treatment with the test drug was considered superior to the comparator in symptomatic relief of dysuria if the lower limit of the 95% CI of the difference between groups was greater than the superiority margin.

For categorical variables, a categorical change test, such as McNemar’s test, was applied to compare patients' notes (0 h, 12 h, 24 h, 48 h, and 72 h; comparison within each treatment). The Chi-squared test was used to test the significance of differences in proportions between drugs (test and comparator). The Kaplan–Meier method and the log-rank test assessed the time to relieve dysuria. Episodes of AEs were analyzed descriptively.

## Results

**Fig. 1:**
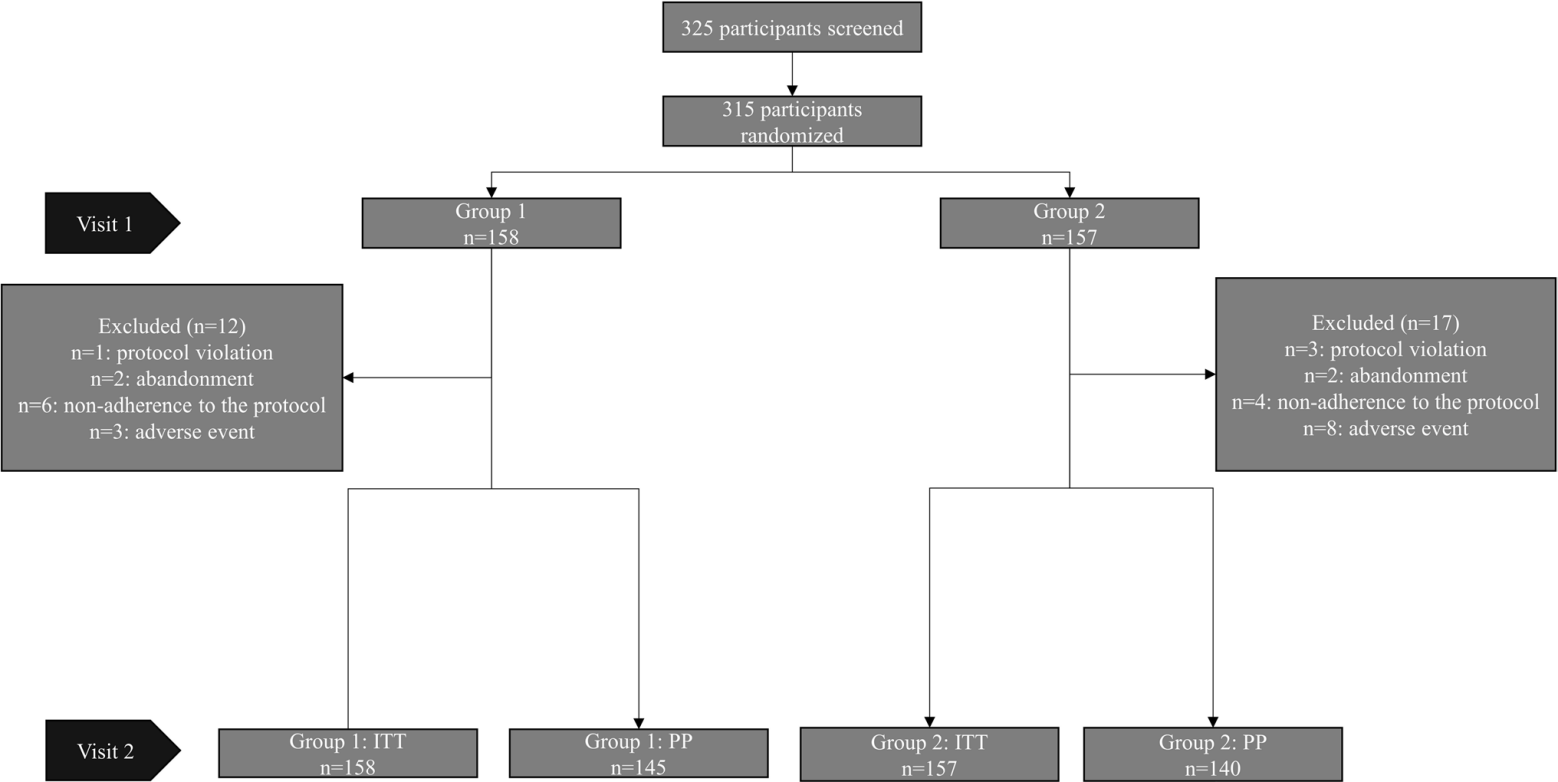
Flow diagram of the study population. *ITT* intention to treat, *PP* per protocol

Three hundred and twenty-five participants were screened, 315 were randomized; 158 participants in group 1 and 157 in group 2 comprised the ITT population. Figure 1 shows the flow diagram of the study population.

Most participants were women (75.9%). The studied population was homogeneously distributed, and symptoms of dysuria were comparable at baseline, without any significant difference (Tables 1, 2).

**Table 1:** Demographics characteristics of the study population

	Group 1 (*n* = 158)	Group 2 (*n* = 157)	*p* value
Women, %	77.2	74.5	0.60
Age, mean years ± SD	53.76 ± 17.38	53.76 ± 17.38	0.59
Height, mean cm ± SD	162.40 ± 7.64	162.14 ± 8.77	0.776
Weight, mean kg ± SD	71.19 ± 16.01	71.07 ± 14.85	0.896
Body temperature, mean °C ± SD	36.21 ± 0.35	36.23 ± 0.35	0.545
Heart rate, mean bpm ± SD	72.06 ± 7.61	71.29 ± 7.05	0.348
Smoking, %	1.9	1.3	0.549
Alcohol abuse, %	5.7	5.7	0.607
Previous surgery, %	12	12.1	1.00
Use of concomitant medications, %	44.9	41.4	0.509

*SD* standard deviation

**Table 2:** Evaluation of dysuria at baseline

	Group 1 (*n* = 158)	Group 2 (*n* = 157)	*p* value
Pain, %			
Absent	0.6	0.0	0.171
Mild	0.0	0.6	
Moderate	97.5	99.4	
Severe	1.9	0.0	
VAS, mean in cm ± SD	5.36 ± 0.84	5.39 ± 0.75	0.756
Burning, %			
Absent	0.6	0.0	0.570
Mild	0.6	0.6	
Moderate	96.8	98.7	
Severe	1.9	0.6	
VAS, mean in cm ± SD	5.45 ± 0.91	5.51 ± 0.80	0.503
Burning on urination, %			
Absent	1.3	0.0	0.507
Mild	0.6	1.3	
Moderate	96.8	97.4	
Severe	1.3	1.3	
VAS, mean in cm ± SD	5.42 ± 0.90	5.52 ± 0.96	0.342

*bpm* beats per minute, *VAS* visual analogue scale, *SD* standard deviation

### Primary outcome

Groups 1 and 2 showed statistically significant improvement in the three symptomatic components of dysuria within 24 h of treatment evaluated with the categorical scale. They were 15.19% in group 1 without pain, 12.03% without burning, and 15.19% without burning on urination versus 3.21%, 3.21%, and 2.56% in group 2 respectively (Table 3).

**Table 3:** Evaluation of dysuria: pain, burning, and burning on urination within 24 h

	Group 1 (*n* = 158)	Group 2 (*n* = 157)	*p* value
Pain, %			
Without improvement	5.06	3.21	0.002
Absent	15.19	3.21	
Mild	13.92	18.59	
Moderate	65.82	75.0	
Burning, %			
Without improvement	5.06	3.21	0.019
Absent	12.03	3.21	
Mild	20.25	20.51	
Moderate	62.66	73.08	
Burning on urination, %			
Without improvement	5.06	3.21	0.001
Absent	15.19	2.56	
Mild	19.62	23.72	
Moderate	60.13	70.51	

In group 1, 16% of participants presented excellent clinical response for pain within 24 h, versus 3.31% in group 2. The difference between groups was 12.69% (95% CI 6.16, 19.21%). In the evaluation of burning, 12.67% of group 1 showed excellent clinical response, versus 3.31% in group 2, a difference of 9.36% (95% CI 3.32, 15.39%). 15.33% of group 1 showed an excellent clinical response in burning on urination versus 2.65% in group 2, a difference of 12.68% (95% CI 6.37, 18.99%). As the lower limit of 95% CI of the difference in the excellent response between the two treatments was greater than 1.85% in all components of dysuria, the test drug was superior to the comparator.

The evaluation of primary outcome in the per protocol (PP) population showed similar results for the three components: pain (95% CI 6.63%, 20.66%), burning (95% CI 3.54%, 16.55%), and burning on urination (95% CI 6.87%, 20.43%).

### Secondary outcomes

The evaluation of excellent clinical response obtained at 12 h, 36 h, 48 h, and 72 h showed that the test drug was superior in pain, burning, and burning on urination from 36 to 48 h (Table 4). At 72 h, the difference between groups was no longer statistically significant (Table 4).

**Table 4:** Evaluation of excellent clinical response at 12 h, 26 h, 48 h, and 72 h

Time	Symptom	Group 1 (*n* = 158), %	Group 2 (*n* = 157), %	Difference, %	95% CI, %	*p* value
12 h	Pain	4.61	2.67	1.94	-2.27, 6.15	0.370
	Burning	3.29	1.33	1.96	−1.42, 5.33	0.260
	Burning on urination	3.95	2.67	1.28	−2.75, 5.31	0.536
36 h	Pain	17.11	4.03	13.08	6.32, 19.84	0.000
	Burning	15.13	3.36	11.78	5.39, 18.16	0.000
	Burning on urination	19.08	6.04	13.04	5.72, 20.36	0.001
48 h	Pain	22.52	11.49	11.03	2.62, 19.44	0.011
	Burning	22.52	11.49	11.03	2.62, 19.44	0.011
	Burning on urination	27.81	14.19	13.63	4.53, 22.72	0.004
72 h	Pain	54.93	45.77	9.15	−2.43, 20.74	0.124
	Burning	55.63	52.82	2.82	−8.77, 14.40	0.635
	Burning on urination	58.45	53.52	4.93	−6.60, 16.46	0.405

Evaluating the time to reach the absence of symptoms according to patients’ reports, 52.41% of group 1 achieved complete resolution of pain within 72 h, and 47.59% were censored, versus 49.28% of group 2 without pain and 50.71% censored. The mean time for complete resolution of pain was 59.42 h (95% CI 56.32 h, 62.52 h) in group 1 and 64.63 h (95% CI 62.52 h, 66.74 h) in group 2, log-rank *p* value = 0.2858. For burning, 55.17% of group 1 had complete resolution within 72 h, and 44.83% were censored. In group 2, 55% had complete resolution, and 45% were censored. The mean time for complete healing of burning was 60.08 h (95% CI 57.22 h, 62.94 h) in group 1 and 64.29 h (62.30 h, 66.27 h) in group 2, log-rank *p* value = 0.5232. 57.93% of group 1 had complete resolution on burning on urination within 72 h, and 42.07% were censored. There were 57.14% in group 2 with complete resolution and 42.86% censored. The mean time for complete healing of burning on urination was 58.01 h (95% CI 54.96 h, 61.07 h) in group 1 and 63.51 h (95% CI 61.28 h, 65.75 h) for group 2, log-rank *p* value = 0.3235.

In the evaluation of sustained healing, 54.93% of group 1 had no pain after 72 h, versus 45.77% of group 2, *p* = 0.124. There were 55.63% in group 1 and 52.82% in group 2 without burning, *p* = 0.635. 58.45% in group 1 had no burning on urination after 72 h versus 53.52% in group 2, *p* = 0.405.

**Fig. 2:**
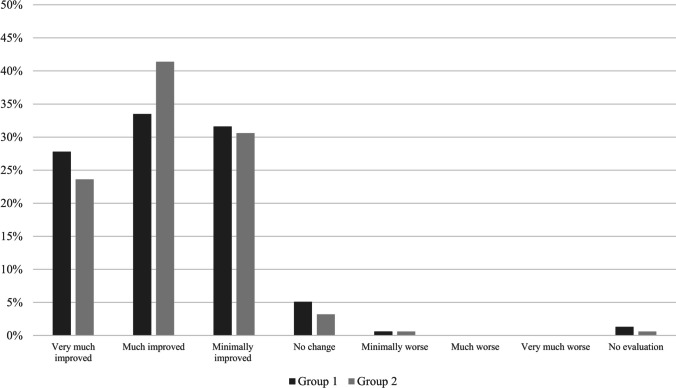
Investigator's evaluation of dysuria according to the Clinical Global Impression scale

According to the investigator's opinion, 61.3% of group 1 and 65.0% of group 2 were categorized as very much to much improved, *p* = 0.768. The proportion of no or minimal improvement and worsening of symptoms was also similar between groups (Fig. 2).

The results with the PP population are also similar for secondary outcomes (data not shown).

Seventy-nine percent of participants in group 1 and 87.8% of group 2 had negative urine culture at V1. At V2, 93.3% of participants in group 1 and 94.8% in group 2 had negative results; 13% of group 1 and 31.3% of group 2 with *Escherichia coli* at V1 presented a negative result at V2. Forty-eight participants, 29 in group 1 (18.35%) and 19 in group 2 (12.10%), had a positive result at V2 and were treated with antibiotic.

### Safety

During the trial, 40 episodes of AEs were recorded: 13 in group 1 and 27 in group 2. The AEs were mild (72.5%) or moderate (27.5%) in intensity, probably to possibly related to treatment, and all of them had complete resolution. The main AEs were nausea/feeling sick: 38.5% of group 1 and 40.7% of group 2; epigastric pain: none in group 1 and 22.2% of group 2; and vomiting: 15.4% of group 1 and 11.1% of group 2. Three participants from group 1 and 8 from group 2 were excluded from the study because of AEs; these events included nausea, vomiting, epigastric pain, dizziness, headache, and malaise.

## Discussion

In this study, the findings were consistent with the hypothesis as the association of methenamine with methylthioninium chloride was superior to phenazopyridine in the treatment of dysuria, shown by a higher percentage of participants with an excellent response rate (improvement of symptoms from mild, moderate, or severe to absent) within 24 h of treatment and the lower limit of the 95% CI for the difference between treatments was greater than the established margin.

Methenamine has been used to prevent uncomplicated lower UTIs as an alternative to antibiotics [16]. Its association with methylthioninium is efficient in the prophylaxis of UTI recurrence and in the symptomatic treatment of UTI before antibiotic therapy, including pain relief on urination [17, 18]. However, no previous study compared this combination with phenazopyridine in dysuria in general, not specifically in UTI. Studies evaluating phenazopyridine in urinary symptoms showed that it efficiently relieves dysuria and the burning sensation of UTI and reduces discomfort from cystoscopy with a similar response to lidocaine [19]. It is comparable with celecoxib and oxybutynin in reducing dysuria in patients under Bacillus Calmette–Guérin therapy [20].

In our study, besides the superiority in relief within 24 h, the improvement in favor of the test drug extended up to 48 h but was no longer significant at 72 h. These findings are in accordance with the literature as a significant improvement after phenazopyridine treatment is observed within 24 h and an absence of symptoms within 72 h [19]. The investigators’ evaluation did not identify a significant difference between groups, as more than 50% of participants had improved pain, burning, and burning on urination from much to very much on the CGI scale in both groups.

Even though UTI was not the focus of this study, but is usually accompanied by dysuria, we decided to explore the bactericidal properties of the test drug as a secondary criterion. Participants with a positive test at V1 did improve with the test drug, especially those positive for the most frequent bacteria found (*Escherichia coli*). This result was in accordance with the literature, as it is reported that methenamine is effective against Gram-positive, Gram-negative, and anaerobic bacteria [21], and that methylthioninium chloride has broad-spectrum bactericidal activity comparable with antibiotics [22].

Regarding the safety of the studied product, a Cochrane review of methenamine hippurate for the prevention of UTIs as well as studies with the association with methylthioninium chloride in the prevention of recurrent and uncomplicated UTIs reported a low rate of AEs, with gastrointestinal disturbances, such as nausea and diarrhea, being the most common [16–18]. In our study, findings were comparable with those in the literature, as the most frequent AEs were gastrointestinal symptoms, including nausea and vomiting; epigastric pain was frequent but only in the phenazopyridine group. No serious AEs were observed during the study.

To our knowledge, this was the first randomized clinical trial to compare the combination of methenamine and methylthioninium chloride with phenazopyridine, providing evidence for a more efficient option in treating dysuria, a frequent symptom in daily practice. However, our study is limited by its single-blind design, as both treatments present a specific coloration in urine, precluding a double-blind scenario, and by its subjective evaluation, being susceptible to observer bias. Further investigations may improve evidence with objective assessments and evaluation of methenamine with methylthioninium in specific conditions causing dysuria.

In conclusion, dysuria is a common complaint in ED worldwide, with limited symptomatic treatments available. Methenamine with methylthioninium chloride is an effective option to quickly resolve dysuria, a well-tolerated treatment, and superior to phenazopyridine.

## Data Availability

The dataset supporting the conclusions of this article is available upon request, by contacting the corresponding author.
